# Event-related potential evidence for tactile orientation processing in the human brain

**DOI:** 10.1007/s00221-024-06783-1

**Published:** 2024-02-24

**Authors:** Jiajia Yang, Rongxia Ren, Yinghua Yu, Wu Wang, Xiaoyu Tang, Yoshimichi Ejima, Jinglong Wu

**Affiliations:** 1 Graduate School of Interdisciplinary Science and Engineering in Health Systems,, Okayama University, 3-1-1 Tsushima-Naka, Kita-ku, Okayama, 700-8530 Japan; 2 Multisensory Laboratory, School of Psychological and Cognitive Sciences, Peking University, Beijing, 100871 China; 3 School of Psychology, Liaoning Collaborative Innovation Center of Children and Adolescents Healthy Personality Assessment and Cultivation, Liaoning Normal University, Dalian, 116029 China

**Keywords:** Somatosensory system, Tactile orientation, Adaptation, Event-related potentials

## Abstract

It is well known that information on stimulus orientation plays an important role in sensory processing. However, the neural mechanisms underlying somatosensory orientation perception are poorly understood. Adaptation has been widely used as a tool for examining sensitivity to specific features of sensory stimuli. Using the adaptation paradigm, we measured event-related potentials (ERPs) in response to tactile orientation stimuli presented pseudo-randomly to the right-hand palm in trials with all the same or different orientations. Twenty participants were asked to count the tactile orientation stimuli. The results showed that the adaptation-related N60 component was observed around contralateral central-parietal areas, possibly indicating orientation processing in the somatosensory regions. Conversely, the adaptation-related N120 component was identified bilaterally across hemispheres, suggesting the involvement of the frontoparietal circuitry in further tactile orientation processing. P300 component was found across the whole brain in all conditions and was associated with task demands, such as attention and stimulus counting. These findings help provide an understanding of the mechanisms of tactile orientation processing in the human brain.

## Introduction

Tactile sensory processing involves neural mechanisms that extract geometric features. The orientation of local edges is a critical feature for tactile object perception. In the somatosensory cortex, the orientation-selectivity of neurons has been extensively studied (Hsiao et al. 2002; Thakur et al. 2006). For example, previous studies (Bensmaia et al. 2008a; Peters et al. 2015a) have shown that neurons in the primary somatosensory cortex  (S1, specifically in area 3b) exhibit robust tuning of stimulus orientation in terms of firing rate. Subsequently, after integrating information from several low-level inputs, the information is thought to be transmitted to higher-level areas, including areas 1 and 2, the secondary somatosensory cortex (S2), the prefrontal cortex (PFC), and the posterior parietal cortex (PPC), for further processing (Sathian 2016). However, the temporal characteristics of tactile orientation processing remain unknown.

Event-related potentials (ERPs), a technique known for its high temporal resolution, are valuable for providing precise temporal information about the neural responses involved in tactile processing. Early ERP components are believed to be involved in the processing of stimulus features, and later components are associated with the perceptual and cognitive processing of stimuli (Thierry 2005; Schubert et al. 2006; Banaschewski and Brandeis 2007; Bolton and Staines 2012). In general, the early ERP components from tactile stimuli include P50 and N80, most likely generated in the contralateral S1. After approximately 100 ms, S2, posterior parietal and frontal regions activate, with the N120 generated in bilateral S2 and the P300 generated bilaterally in increasingly parietal areas beyond somatosensory regions. Given the sensitivity of somatosensory ERP components, this approach provides an ideal means of exploring tactile orientation processing. To date, a thorough investigation into tactile orientation processing has not been undertaken.

Adaptation occurs after exposure to multiple repetitions of the same or similar stimulus without intervening items (Simons et al. 2003; Harvey and Burgund 2012), which results in a reduced neural response to repeated stimuli compared with responses to unrepeated stimuli (Henson and Rugg 2003; Noguchi et al. 2005; Tamè et al. 2015). Adaptation has been widely used as a tool to study sensitivity to specific features of sensory stimuli. If the underlying neural representation is insensitive to the change in the stimulus, then the signal will be reduced, similar to the reduction produced by repetitions of identical stimuli. Alternatively, if the neurons are sensitive to the transformation, the signal will return to the original (non-adopted) level (Grill-Spector et al. 2006). Previous studies have shown that many features of tactile stimuli, such as curvature (Van der Horst et al. 2008) and distance (Calzolari et al. 2017), are related to susceptibility to adaptation. Thus, ERPs combined with adaptation can provide an experimental probe for investigating whether tactile orientation characteristics result from relatively earlier or later stages of somatosensory processing.

In the present study, we used a custom-designed piezo device (Braille pattern stimulus) to present the tactile orientation stimulus to participants’ right hand with a 2000–2500 ms interval, and the participants were asked to count the number of two series of orientations (i.e., twelve same orientations or different orientations). By comparing the ERPs evoked separately by the first, third, sixth, and ninth presentations of a recurrent somatosensory orientation stimulus, we tested how tactile orientation adaptation modulates brain activity over time. We expected to find reduced ERP components due to the adaptation effect for the same orientation series condition, while similar suppression would disappear for the different orientation series conditions at the whole-brain level.

## Materials and methods

### Participants

A power analysis using G*Power (Faul et al. 2009) established that the experiments were properly powered to detect an effect [parameters: η_p_^2^ = 0.19 (Tamè et al. 2015); effect size f = 0.48; power = 0.80]. A minimum of 8 participants is required. During ERP experiment, participant data are prone to being eliminated due to artifacts. Ultimately, twenty participants (age range: 22–25 years; *M* = 22.75 years, *SD* = 1.05; all male), three of whom were left-handed, participated in the experiment. None of the participants reported any loss of tactile sensation or a history of major medical or neurological illness, such as epilepsy, significant head trauma, or alcohol dependence. The participants provided written, informed consent prior to the experiment. The experimental protocol was approved by the local Medical Ethics Committee at Okayama University Hospital. All methods were carried out in accordance with the approved guidelines.

### Stimuli

**Fig. 1:**
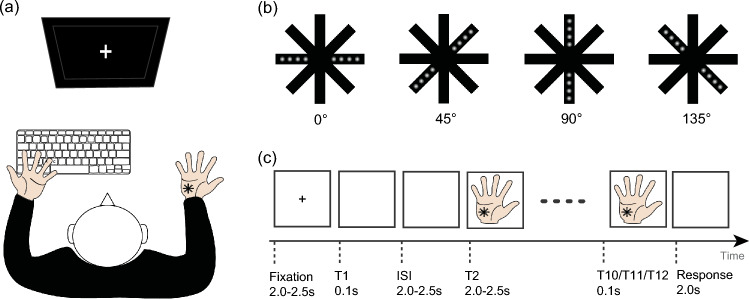
Illustration of the experimental setup, the tactile stimuli, and an example trial. **a** Experimental setup. **b** Tactile orientation stimuli. **c** The sequence of events and their durations, which are independent of whether a trial was the same or different orientation condition. The participants were asked to count the tactile stimulation by using the response key during a 2-s response phase

The experiment was conducted in a dimly illuminated, sound-attenuated room. The participants were seated directly in front of a computer monitor (Fig. 1a), with their heads held steady by a chin rest. Tactile stimuli were presented to the right palm using a custom-designed piezo-electric stimulator (Piezostimulator, SC9 equipment, KGS Company, Japan). As shown in Fig. 1b, a total of four orientation stimuli (i.e., 0°, 45°, 90° and 135°) were used, and each orientation stimulus consisted of eight plastic pins. Pins could be elevated from the resting position by 0.7 mm with a rise-time of 1 ms [tactile force = 0.177 N]. The pin diameter is 1.3 mm.

### Procedure and design

The experimental procedure is illustrated in Fig. 1c. Each trial began with participants fixating on a white cross in the center of the screen for 2.0–2.5 s. Following the fixation stimulus, 0.1 s tactile orientation stimuli were presented to the right-hand palm. Then, no stimulus was presented during the inter-stimulus interval (ISI), which was assigned a random duration between 2.0 and 2.5 s. Next, the second tactile orientation stimulus was presented for 0.1 s. After presenting 10, 11 or 12 tactile orientation stimuli, the trial ended, and the participants were instructed to complete the count task by pressing the reaction keys with their left hand.

Two factors, orientation and stimulus order, were manipulated in the experiment. The orientation condition had two levels: the same and different orientations. In the same orientation trials, all the tactile orientation stimuli were presented in the same orientation, 45° or 135°. In different orientation trials, stimuli with 0°, 45°, 90°, and 135° orientations were presented in a pseudorandom order. There were equal numbers of trials with the same orientation and trials with different orientations. After completing one practice block, each participant completed 20 experimental blocks in a pseudorandom order for a total of 200 trials. Thus, there were 100 trials for each experimental condition. Only the ERP responses to the 1st, 3rd, 6th, and 9th stimuli in a trial are reported.

### ERP data recording and analysis

EEG data were recorded using a 32-channel EEG system (Brainamp™ amplifiers) with active electrodes (Acticap™) and software (Brain Vision Recorder 2.0), all of which were manufactured by Brain Products (Germany). The electrodes were mounted according to the 10–20 International System, initially referenced to the left mastoid (M1) and then re-referenced to the right mastoid (M2). A ground electrode was incorporated into the cap on the medial frontal aspect. Electrode impedances were kept under 5 KΩ. A horizontal electrooculogram (EOG) was recorded from the outer canthi of the left eye, and a vertical EOG was recorded from an electrode placed 1.5 cm above and below the left eye. The signals were amplified, digitized, and filtered using a sampling rate of 500 Hz.

The data analysis was performed using the Brain Vision Analyzer. The data were digitally filtered with a high-pass filter with a 0.1 Hz cutoff and a low-pass filter with a 30 Hz cutoff (24 dB/octave). The data were segmented in 600 ms long epochs from − 100 to 500 ms. Artifact rejection was performed using a semiautomated procedure to remove EEG epochs that included eye movements and blinks. Additionally, trials with excessive artifacts (± 100 μV) were excluded from further analysis. Eventually, 13.39% of the trials were eliminated. The data from each electrode were averaged, and a grand average ERP was computed across all participants for each experimental condition using the R package.

**Fig. 2:**
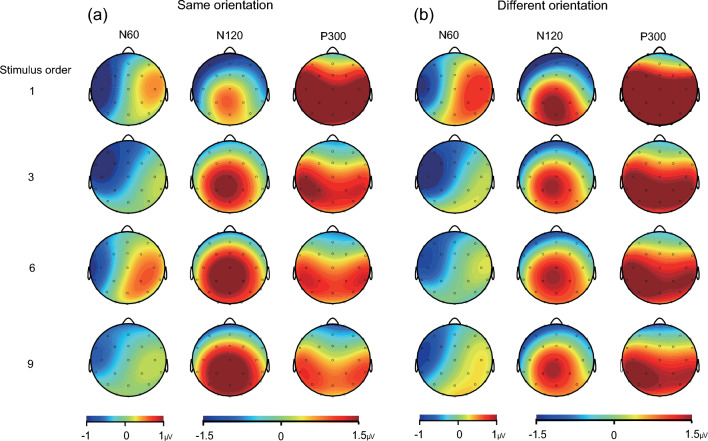
Topographic maps for the N60 (50–70 ms), N120 (100–150 ms) and P300 (230–330 ms) responses to tactile stimuli delivered in the same orientation (**a**) and different orientation (**b**) conditions

Figure 2 shows scalp topographic maps at three time points. First, at 60 ms, there was strong negativity across the contralateral central-parietal cluster (C3, CP5, T7 electrodes), which overlies the somatosensory cortex. Second, distributed negative activity across ipsilateral and contralateral frontal sites (FC5/6, F7/8 electrodes) occurred at 120 ms. These electrodes were close to and over somatosensory areas, similar to the selection of electrodes in previous somatosensory ERP studies (Forster et al. 2009; Jones and Forster 2012; Novičić and Savić 2023). Third, the scalp topography also showed that the parietal lobe area was broadly distributed (P3/4, P7/8 electrodes) at approximately 300 ms, which is responsible for tactile perception according to Gottlieb (2007) and (Chen et al. 2019). Three clear somatosensory ERP components were identifiable from the grand averages: an N60 component in the 50–70 ms time window, an N120 component in the 100–150 ms time window and a P300 component between 230 and 330 ms.

Statistical analyses of N60, N120, and P300 were also performed using the R package. Mean amplitude values were analyzed separately for each time window by separate three-way repeated-measures ANOVAs for factor orientation (same and different), stimulus order (1st, 3rd, 6th, and 9th stimulus in the series) and hemisphere (contralateral versus ipsilateral to stimulated hand). Latency values were analyzed by separate two-way repeated-measures ANOVAs for factor orientation (same and different) and hemisphere (contralateral versus ipsilateral to stimulated hand). The Maulchy test was used to examine the sphericity of the data, and the Huynh–Feldt epsilon correction was applied when the sphericity assumption was violated. In the latter case, the epsilon was reported together with the corrected *p* value and corrected degrees of freedom. Significant effects were investigated by post hoc Tukey’s multiple comparisons tests. The statistical significance level was set to *p* < 0.05 after correction.

## Results

### N60

#### N60 amplitude (50–70 ms)

As shown in Fig. 2, N60 appeared near the contralateral central-parietal area. Two-way repeated-measures ANOVAs for factor orientation (same and different) and stimulus order (1st, 3rd, 6th, and 9th stimulus in the series) were subsequently conducted. The analysis of the N60 amplitude revealed no main effects of stimulus order [*F* (2.85, 54.12) = 2.06,* p* = 0.119, η^2^_p_ = 0.098] or orientation [*F* (1, 19) = 0.173,* p* = 0.682, η^2^_p_ = 0.009]. In addition, there was no interaction between stimulus order and orientation [*F* (2.76, 52.46) = 2.03,* p* = 0.126, η^2^_p_ = 0.097]. Follow-up analysis separated for the same orientation and different orientation conditions.

**Fig. 3:**
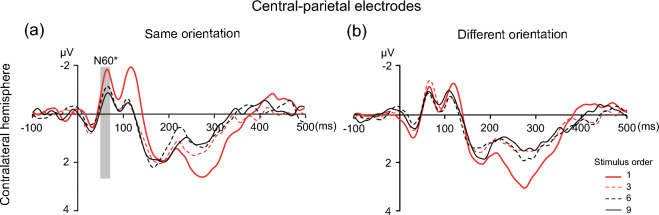
ERP waveforms at central-parietal electrodes time-locked to the onset of the first, third, sixth and ninth tactile stimuli for the same (**a**) and different orientation (**b**) conditions. The time windows used to measure the N60 amplitude (50–70 ms) are denoted by the shaded areas. ^*^p < 0.05, ^**^p < 0.01, ^***^p < 0.001

In the same orientation condition (see Fig. 3a), a one-way repeated-measures ANOVA including the N60 amplitudes of the 1st, 3rd, 6th, and 9th stimuli demonstrated a significant main effect of stimulus order [*F* (3, 57) = 3.02,* p* = 0.037, η^2^_p_ = 0.137] near the contralateral S1, indicating that the adaptation effect occurred. Post hoc tests showed that the mean amplitude of the N60 component was significantly enhanced for the 1st stimulus (M_1st_ = − 1.494, SE = 0.35) than for the 9th stimulus (M_9th_ = − 0.633, SE = 0.21, *t* (19) = 3.03, *p* = 0.0319, Cohen’s *d* = 0.65); however, other comparisons did not yield significant differences.

In different orientation condition (see Fig. 3b), one-way repeated-measures ANOVA including the N60 amplitudes of the 1st, 3rd, 6th, and 9th stimuli demonstrated a nonsignificant main effect of stimulus order near the contralateral central-parietal area [*F* (3, 57) = 0.96,* p* = 0.42, η^2^_p_ = 0.048], indicating that the adaptation effect did not occur.

### N120

#### N120 amplitude (100–150 ms)

**Fig. 4:**
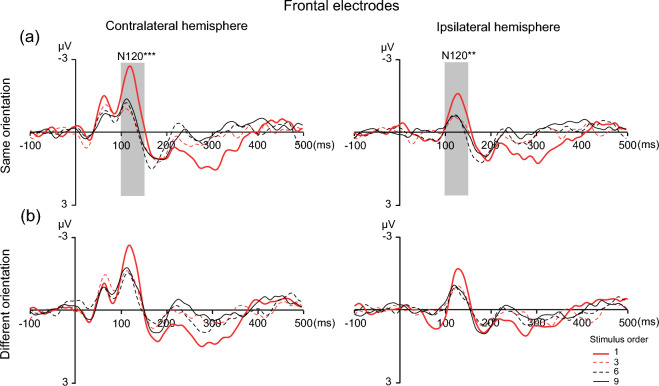
ERP waveforms at frontal electrodes time-locked to the onset of the first, third, sixth and ninth tactile stimuli for the same (a) and different (b) orientation conditions. The time windows used to measure the N120 amplitude (100–150 ms) are denoted by the shaded areas. ^*^p < 0.05, ^**^p < 0.01, ^***^p < 0.001

As shown in Fig. 4, the analysis of the N120 amplitude revealed a main effect of stimulus order [*F* (2.70, 51.32) = 13.45,* p* < 0.001, η^2^_p_ = 0.414], confirming that ERP amplitudes decreased as the stimulus order increased. The main effects of orientation [*F* (1, 19) = 7.166,* p* = 0.015, η^2^_p_ = 0. 274] and hemisphere [*F* (1, 19) = 12.897,* p* = 0.002, η^2^_p_ = 0.404] were also significant. In addition, there were no three-way interactions [stimulus order × orientation × hemisphere: *F* (2.236, 42.481) = 0.752,* p* = 0.492, η^2^_p_ = 0.038] or two-way interactions [stimulus order × orientation: *F* (2.672, 50.767) = 0.886,* p* = 0.445, η^2^_p_ = 0.045; stimulus order × hemisphere: *F* (2.082, 39.564) = 2.080,* p* = 0.137, η^2^_p_ = 0.099; orientation × hemisphere: *F* (1, 19) = 2.915,* p* = 0.104, η^2^_p_ = 0.133]. Follow-up analysis was performed separately for the same orientation and different orientation conditions.

In the same orientation condition (see Fig. 4a), one-way repeated-measures ANOVA of the N120 amplitudes of the 1st, 3rd, 6th, and 9th stimuli demonstrated a significant main effect of stimulus order [*F* (3, 57) = 10.81,* p* < 0.001, η^2^_p_ = 0.363] at the contralateral sides of the frontal cortex, indicating that the adaptation effect occurred. Post hoc tests showed that the mean amplitude of the N120 component was significantly greater for the 1st stimulus (M_1st_ = − 1.853, SE = 0.293) than for the 3rd (M_3rd_ = − 0.454, SE = 0.159, *t* (19) = 4.946, *p* < 0.001, Cohen’s *d* = 1.177), 6th (M_6th_ = − 0.469, SE = 0.267, *t* (19) = 4.629, *p* = 0.001, Cohen’s *d* = 1.164) and 9th (M_9th_ = − 0.668, SE = 0.203, *t* (19) = 4.165, *p* = 0.0027, Cohen’s *d* = 0.997) stimuli, while other comparisons did not yield significant differences. On the ipsilateral sides of the frontal cortex, a significant main effect of stimulus order [*F* (3, 57) = 3.41,* p* = 0.023, η^2^_p_ = 0.152] was revealed. Post hoc tests showed that the mean amplitude of the N120 component was significantly greater for the 1st stimulus (M_1st_ = − 1.052, SE = 0.245) than for the 6th stimulus (M_6th_ = − 0.360, SE = 0.259, *t* (19) = 3.543, *p* = 0.011, Cohen’s *d* = 0.582); however, other comparisons did not yield significant differences.

In different orientation conditions (see Fig. 4b), one-way repeated-measures ANOVA on the N120 amplitudes of the 1st, 3rd, 6th, and 9th stimuli demonstrated a significant main effect of stimulus order at the contralateral frontal cortex [*F* (3, 57) = 3.26,* p* = 0.028, η^2^_p_ = 0.147]. However, no comparisons yielded significant differences. On the ipsilateral sides of the frontal cortex, a main effect of stimulus order [*F* (3, 57) = 2.68,* p* = 0.056, η^2^_p_ = 0.123] was not revealed.

### N120 latency

As shown in Table 1, the analysis of N120 latency revealed the main effects of hemisphere [*F* (1, 19) = 47.54,* p* < 0.001, η^2^_p_ = 0.714] and orientation [*F* (1, 19) = 7.87,* p* = 0.011, η^2^_p_ = 0.293]. No interaction between orientation and hemisphere was found [*F* (1, 19) = 0.008,* p* = 0.931, η^2^_p_ < 0.001]. Follow-up analysis was performed separately for the same orientation and different orientation conditions.

**Table 1:** The latency (in ms) of the N120 and P300 phases

Orientation	Hemisphere	Component	
		N120	P300
Same	Contralateral	116	268
	Ipsilateral	124	272
Different	Contralateral	118	274
	Ipsilateral	122	274

In the same orientation condition, a paired *t* test comparing the N120 latencies of the ipsilateral and contralateral frontal cortex revealed a significant main effect of hemisphere [*t* (19) = − 5.79,* p* < 0.001, Cohen’s *d* = 1.183], indicating that the tactile orientation N120 latency of the contralateral frontal cortex (M = 113 ms, SE = 1.21) was significantly shorter than that of the ipsilateral frontal cortex (M = 122 ms, SE = 1.92).

In different orientation condition, the main effect of hemisphere [*t* (19) = − 5.28,* p* < 0.001, Cohen’s *d* = 1.206] was also significant, showing that the tactile orientation N120 latency of the contralateral frontal cortex (M = 117 ms, SE = 2.11) was significantly shorter than that of the ipsilateral frontal cortex (M = 126 ms, SE = 2.02).

### P300

#### P300 amplitude (230–330 ms)

**Fig. 5:**
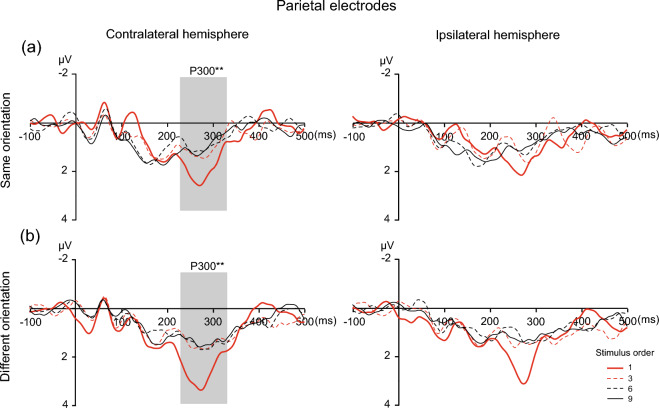
ERP waveforms at parietal electrodes time-locked to the onset of the first, third, sixth and ninth tactile stimuli for the same (**a**) and different (**b**) orientation conditions. The time windows used to measure the P300 amplitude (230–330 ms) are denoted by the shaded areas. ^*^p < 0.05, ^**^p < 0.01, ^***^p < 0.001

As shown in Fig. 5, the analysis of the P300 amplitude revealed a main effect of stimulus order [*F* (1.526, 29.001) = 8.656,* p* = 0.002, η^2^_p_ = 0.313], confirming that ERP amplitudes decreased as the stimulus order increased. The main effects of orientation [*F* (1, 19) = 8.208,* p* = 0.010, η^2^_p_ = 0. 302] were significant. No main effect of hemisphere [*F* (1, 19) = 3.410,* p* = 0.080, η^2^_p_ = 0.152] was found. In addition, there were no three-way interactions [stimulus order × orientation × hemisphere: *F* (2.639, 50.138) = 0.756,* p* = 0.508, η^2^_p_ = 0.038] or two-way interactions [stimulus order × orientation: *F* (2.272, 43.166) = 0.318,* p* = 0.756, η^2^_p_ = 0.016; stimulus order × hemisphere: *F* (2.263, 42.988) = 2.122,* p* = 0.126, η^2^_p_ = 0.10; orientation × hemisphere: *F* (1, 19) = 1.809,* p* = 0.194, η^2^_p_ = 0.087]. Follow-up analysis was performed separately for the same orientation and different orientation conditions.

In the same orientation condition (Fig. 5a), one-way repeated-measures ANOVA on the P300 amplitude of the 1st, 3rd, 6th, and 9th stimuli demonstrated a significant main effect of stimulus order [*F* (3, 57) = 6.01,* p* = 0.001, η^2^_p_ = 0.240] on the contralateral side of the parietal cortex, indicating that an adaptation effect occurred. Post hoc tests showed that the mean amplitude of the P300 component was significantly greater for the 1st stimulus (M_1st_ = 1.91, SE = 0.297) than for the 6th stimulus (M_6th_ = 0.943, SE = 0.221, *t* (19) = − 3.018, *p* = 0.033, Cohen’s *d* = − 0.717) or 9th stimulus (M_9th_ = 0.935, SE = 0.168, *t* (19) = − 4.273, *p* = 0.0021, Cohen’s *d* = − 0.723); however, other comparisons did not yield significant differences. At the ipsilateral parietal cortex, a significant main effect of stimulus order was revealed [*F* (3, 57) = 3.15,* p* = 0.03, η^2^_p_ = .142]. However, no comparisons yielded significant differences.

In different orientation condition (see Fig. 5b), one-way repeated-measures ANOVA on the P300 amplitudes for the 1st, 3rd, 6th, and 9th stimuli demonstrated a significant main effect of stimulus order [*F* (3, 57) = 6.11,* p* = 0.001, η^2^_p_ = 0.243] on the contralateral sides of the parietal cortex, indicating that a tactile orientation adaptation effect occurred. Post hoc tests showed that the mean amplitude of the P300 component was significantly greater for the 1st stimulus (M_1st_ = 2.634, SE = 0.453) than for the 3rd stimulus (M _3rd_ = 1.447, SE = 0.254, *t* (19) = − 2.981, *p* = 0.035, Cohen’s *d* = − 0.88). On the ipsilateral sides of the parietal cortex, there was no significant main effect of stimulus order [*F* (3, 57) = 3.94,* p* = 0.13, η^2^_p_ = 0.172], which indicated that the adaptation effect did not occur.

#### P300 latency

As shown in Table 1, the analysis of P300 latency revealed no main effects of hemisphere [*F* (1, 19) = 0.064,* p* = 0.803, η^2^_p_ = 0.003] or orientation [*F* (1, 19) = 0.078, * p* = 0.784, η^2^_p_ = 0.004]. No interaction between orientation and hemisphere was found [*F* (1, 19) = 0.33,* p* = 0.573, η^2^_p_ = 0.017]. Follow-up analysis was performed separately for the same orientation and different orientation conditions.

For the same orientation condition, paired *t* tests were performed to compare the P300 latencies of the ipsilateral and contralateral parietal cortices. The main effect of hemisphere was not significant [*t* (19) = 0.29,* p* = 0.775, Cohen’s *d* = 0.039], indicating that there was no significant difference between the contralateral parietal cortex (M = 277 ms, SE = 3.66) and the ipsilateral frontal cortex (M = 276 ms, SE = 3.88). In different orientation trials, the main effect of hemisphere [*t* (19) = − 0.45,* p* = 0.656, Cohen’s *d* = 0.117] was also not significant, revealing that there was no significant difference between the contralateral parietal cortex (M = 276 ms, SE = 4.10) and the ipsilateral parietal cortex (M = 278 ms, SE = 3.40).

## Discussion

In the present study, we investigated brain activity during tactile orientation adaptation using an ERP experiment. Our results extended previous findings (Jones and Forster 2012, 2013; Juravle et al. 2016; Gherri et al. 2023; Novičić and Savić 2023) by revealing that the tactile orientation adaptation effect occurred across the whole brain starting from 60 (i.e., N60) to 300 ms (i.e., N120, P300). Furthermore, we found that the adaptation-related N60 component occurred around the contralateral central-parietal areas, which may reflect orientation processing in the somatosensory areas. In contrast, an adaptation-related N120 component was found across the bilateral hemispheres, which suggested that the frontoparietal circuit (Yang et al. 2014) plays a role in further tactile orientation processing. The P300 component, however, was found across the whole brain in all conditions and was related to the task demanded, such as attention and stimulation counting. Together, our findings revealed the existence of an apparent adaptation effect related to tactile orientation processing across the brain from the temporal dimension. These findings help provide an understanding of the mechanisms of tactile orientation processing in the human brain.

We found an initial tactile orientation adaptation effect at approximately 60 ms after stimulus onset around the contralateral central-parietal areas (Fig. 2). According to previous findings (Bensmaia et al. 2008b; Peters et al. 2015), the N60 amplitudes decreased from the first repetition within the same orientation session, which may reflect early orientation processing within the contralateral S1 (Allison et al. 1992). While we cannot refer to the exact source location for the N60 component due to methodological limitations in ERPs, one possibility is that orientation processing within the S1 subregions (i.e., areas 3b, 1, and 2) contributed to the N60 adaptation effect. Both animal (Peters et al. 2015) and human (Yu et al. 2019; Yang et al. 2021) studies have shown that sensory inputs from mechanoreceptive afferents yield activity in the contralateral area 3b via the thalamus, after which the neural signals from area 3b project to areas 1 and 2 for further spatial processing (e.g., edge detection, orientation construction). Thus, the repetition of the same orientation in the present study may attenuate the activation of the cortical circuit across the S1 subregions at the early stage of somatosensory processing, and this attenuation may occur at approximately 60 ms.

We also found an adaptation effect of the N120 component (Fig. 4) in the same orientation conditions across the bilateral hemispheres rather than in the contralateral central-parietal areas. A possible interpretation of this finding is related to hierarchical somatosensory processing in the human brain. When orientation stimuli contact the skin, spatially modulated discharge patterns at the mechanosensory afferent level are evoked (Johansson and Birznieks 2004). Then, this spatial information is projected to areas 3b and 1, and some of the neurons in these areas may participate in the early stage of orientation perception. Subsequently, the information may be assembled into a more complete orientation within S2 and other higher-level regions (i.e., regions involved in the frontoparietal circuit). Therefore, the widespread N120 adaptation effect observed in the present study suggested that these regions lie at a later orientation processing stage than contralateral S1. However, it is still difficult to determine whether the N120 component reflects orientation processing per se or other higher-order functions, such as working memory. For example, a previous study showed that the N120 reflects the ability to extract and maintain information to a certain extent (Bradley et al. 2016). In addition, the peak latency of contralateral N120 activity occurred 10 ms earlier than that of ipsilateral N120 activity (Table 1). Based on possible anatomical pathways (Tamè et al. 2016), the interhemispheric delay of 10 ms may be consistent with callosal transmission (Cracco et al. 1989; Noachtar et al. 1997). In the present study, the participants were asked to count how many stimuli were perceived for each trial for both the same and different orientation conditions. To clarify this point, our future studies will focus on manipulating the experimental factors to better elucidate tactile orientation processing in the human brain.

P300 is an important and extensively explored late component of ERP studies that is widely applied to assess cognitive function in humans. For example, the P300 has been suggested to be a marker of boredom and waning attention (Datta et al. 2007). In the present study, we found a stronger P300 component in response to the first stimulus than in response to all the other stimuli in both the same and different orientation conditions. Thus, some of the factors that contribute to P300 may be identical for the first stimulus regardless of the task. In the present study, to test the orientation adaptation effect, we asked all participants to count the number of stimulation series and did not try to discriminate the orientations. Thus, the task difficulty between the same and different orientation conditions was assumed to be the same. One possibility is paid more attention to the first stimulus in both conditions, which evoked a stronger P300 component than other stimuli. Another possible interpretation of this finding is related to other top-down effects, such as predictions (Kotchoubey et al. 1997; Peng et al. 2012). In line with this view, we could expect that after the first stimulation, participants would predict which type of orientation would be presented. Taken together, even attention and prediction are not mutually exclusive phenomena, and we cannot conclude which factor contributes much to P300; however, our findings indicated that the P300 signature was not related to the orientation adaptation.

To our knowledge, this is the first report of ERP evidence for human tactile orientation processing. ERP correlates of tactile orientation showed early modulation of the N60 component over contralateral S1 areas in the same orientation condition. This early modulation most likely reflects sensitivity to tactile orientation features. The subsequent N120 modulations were presented across the central-parietal region in the same orientation condition. It was predicted to be the most likely component associated with both orientation processing and other higher-order functions, such as working memory. Finally, the widespread P300 component may reflect some sort of top-down processing regardless of the same or different orientation conditions.

## Data Availability

The data will be made available upon request.
